# Pd-Based Membranes: Overview and Perspectives

**DOI:** 10.3390/membranes9020025

**Published:** 2019-02-01

**Authors:** Thijs Peters, Alessio Caravella

**Affiliations:** 1 SINTEF Industry, 0314 Oslo, Norway; 2 Department of Environmental and Chemical Engineering (DIATIC), University of Calabria, Via P. Bucci, Cubo 44A, 87036 Rende (CS), Italy; alessio.caravella@unical.it

**Keywords:** palladium-based membrane, hydrogen, manufacturing, demonstration

## Abstract

Palladium (Pd)-based membranes have received a lot of attention from both academia and industry thanks to their ability to selectively separate hydrogen from gas streams. Integration of such membranes with appropriate catalysts in membrane reactors allows for hydrogen production with CO_2_ capture that can be applied in smaller bioenergy or combined heat and power (CHP) plants, as well as in large-scale power plants. Pd-based membranes are, therefore, regarded as a Key Enabling Technology (KET) to facilitate the transition towards a knowledge-based, low carbon and resource-efficient economy. This Special Issue of the journal *Membranes* on “Pd-based Membranes: Overview and Perspectives” contains nine peer-reviewed articles. Topics include manufacturing techniques, understanding of material phenomena, module and reactor design, novel applications, and demonstration efforts and industrial exploitation.

## 1. Introduction

This Special Issue of *Membranes* focuses on several new aspects of Pd-based membranes for hydrogen separation and their main applications. The papers comprising the Special Issue should be read as single chapters of a whole story, starting from membrane fabrication, passing through materials investigations and technology development, and concluding with the main applications. The next section provides an overview of highlights from each “chapter” reported in order, starting with Pd-membrane manufacturing.

## 2. Highlights of This Special Issue 

### 2.1. Pd-Membrane Manufacturing

Thin state-of-the-art Pd-based membranes in general are being constituted of a thin selective Pd or Pd-alloy layer applied onto a porous support providing for mechanical strength. Dense metal membranes display extremely high levels of selectivity, however, a lower thickness limit seemingly exists for which a dense layer can be obtained [1]. This thickness limit increases with surface roughness and pore size in the support’s top layer [2]. Clearly, this relationship makes strong demands on the pore size distribution of the supports [3,4]. It is, therefore, usual to carry out some form of pre-treatment or surface modification of the support to improve the final quality of the membrane. An overview of such strategies, and available commercial support materials, is accurately described by Alique et al. in this Special Issue [4]. Currently, several techniques for the determination of the pore size distribution of porous supports exist, but there is still a lack of efficient methods for determination of the size of the pore opening [5], as well as of the defect distribution of dense Pd-based composite membranes. In this Special issue of *Membranes*, a novel “modified” liquid–liquid displacement porometry (MLLDP) to quantify the pore opening size is introduced by Zheng et al. [5]. This method can operate under reasonably low pressures for a wide spectrum of pore sizes, due to the relatively lower liquid–liquid interfacial tensions compared to gas–liquid surface tensions. In addition, the “entraining phenomenon” can be eliminated in the MLLDP method. As with other techniques, the accuracy of MLLDP, however, decreases with increasing pore size. In the article, the applicability of the MLLDP method for the measurement of the defect size distribution for Pd composite membranes is also shown. This may be an interesting technique to assess pinhole formation during long-term operation. 

Several technologies can be used to apply a thin layer of Pd or Pd-based alloys, onto a porous support. For the most commonly applied technique, electroless plating, a thorough review of recent developments is presented by Alique et al. [4]. Strategies to improve the deposition itself, e.g., to increase the film homogeneity and to reduce the carbon deposits, are described in this article. Moreover, membrane repair and protection strategies are introduced. In addition, Wunsch et al. present an alternative cost-effective technique for metal-layer deposition by suspension plasma spraying, using supports composed of porous sinter metallic supports made of Crofer-22 APU deposited with a YSZ diffusion-barrier layer [6]. Advantages lie in the short time requirements for deposition, substrates do not have to be activated as in the case of electroless plating, and no metal-loaded liquid waste is produced. Initial results were not satisfactory, however, since deposited layers had remaining open porosity, but work is ongoing [6].

### 2.2. Material Investigations

For metal membranes to be massively integrated into industrial processes to separate hydrogen from gas mixture, it is crucial to assess their performance and stability under actual operating conditions. In particular, as these types of membranes are thought to operate at relatively high temperatures (300–600 °C) and high pressure (2–50 bar and even more), while also in transient conditions in which they can be subjected to rapid changes, it is essential to understand the behavior of the materials they are made of under such conditions.

In this Special Issue, three papers regarding novel and non-conventional studies of materials are offered. In particular, Vicinanza et al. [7] studied the heat treatment of Pd-based membranes, separating the single contributions of both membrane surfaces (i.e., on the feed and permeate side). In their work, they consider three different membrane thicknesses, from whose analysis the effects of adsorption and desorption are disentangled, quantifying the surface phenomena influence, and also in terms of the apparent Arrhenius parameters for permeation before and after the heat treatment [7].

Complementary to the work of Vicinanza et al. [7], Løvvik et al. [8] carried out an interesting and specific work on the influence of grain boundary segregation of bulk in Pd-Ag-Cu membranes, an area to which the literature has generally paid relatively poor attention. Specifically, this study is based on first-principles electronic structure calculations performed on realistic atomic-scale models of binary Pd-Cu and ternary Pd-Cu-Ag alloys. In this way, a systematic approach to designing metal alloys is introduced, which opens up the possibility of more precisely predicting the behaviour of metal lattices a priori, thus reducing the number of experimental tests required and the costs related to the development of new membrane alloy materials. 

In the third paper in this section, Bellini et al. [9] provide an original review on thermodynamic aspects related to hydrogen-metal systems in non-ideal conditions (i.e., pressure-dependent diffusivity and solubility [10,11,12,13]). Analysing information drawn from several studies in the open literature they show a systematic thermodynamic approach based on the chemical potential of the Pd-H system to deal with modeling of hydrogen solubility in the lattice. In particular, an explicit expression for the activity of H atoms in the lattice is obtained, allowing membrane behavior to be modeled under conditions of interest for real industrial applications.

### 2.3. Module and Reactor Design

This Special Issue of *Membranes* would not be complete if it did not provide an insight into novel module and reactor design. Here, micro-membrane reactors that enhance heat management, reduce gas phase diffusion limitations, and increase the membrane area to reactor volume ratio compared to traditional tubular reactors are introduced [14,15]. The papers dealing with this topic are those of Wunsch et al., which report a number of aspects related to micro-membrane reactors, ranging from reactor configuration development, feasible and low-cost fabrication techniques of micro-membranes, and optimal coupling and integration of reactive and separating processes in single compact modules [6,16]. Specifically, Dittmeyer and colleagues at the Institute for Micro Process Engineering at the Karlsruhe Institute of Technology (KIT) have developed micro-membrane reactors composed of stacks of sub-modules including multiple reactive and permeative stages. Even though the idea of using staged membrane reactors is not new [17], the technological approach at KIT is to develop compact systems so as to minimize the drawbacks of larger-scale devices, such as heat removal, concentration polarization and a relatively low membrane surface area per catalyst volume (ca. 10^3^–10^6^ m^−1^). The main applications of these micro devices, for now, are reforming of methane and dehydrogenation of liquid organic hydrogen carriers (LOHCs) [6,16], which we also refer to in Section 2.4.

### 2.4. Applications of Pd-based Membranes

The application of Pd-based membrane technology is currently mainly focused on producing ultrapure hydrogen from fossil sources. As an alternative to hydrogen purification through partial oxidation (PROX) and pressure swing adsorption (PSA), Pd-based membranes have received much attention in the last 30 years because they combine the reforming reaction for hydrogen generation and its separation/purification. The majority of these studies investigate Pd-based membrane reactors in process schemes involving reforming of methane. For example, a 40 Nm^3^/h-class membrane reformer (MRF) system for H_2_ production has been developed by Tokyo Gas, and its long-term durability and reliability have been demonstrated over 8000 h [18,19]. Along the same lines, Pd-based membrane integration is investigated in the fuel processor of distributed combined heating and power (CHP) plants employing fuel cell technology [20], leading to a drastic fuel processing plant simplification.

Many other alternative liquid hydrocarbons and oxygenates may as well be used for the production of hydrogen at a smaller scale in reforming or gasification processes, e.g., methanol, ethanol, glycerol or diesel, originating either from biomass or fossil sources. Among the various renewable fuels, methanol is an interesting hydrogen source because at room temperature it is liquid, and therefore easy to handle and to store. Furthermore, it shows a relatively high H/C ratio and low reforming temperature, ranging from 200 to 300 °C. In their contribution, Iulianelli et al., describe the progress in the last decades with respect to modeling studies on methanol steam reforming in membrane reactors [21].

Pd-based membrane technology is also considered in gas-to-liquid (GTL) processes and chemical synthesis, such as alkane dehydrogenation (DH) reactions. These applications, and their technical feasibility verified at the pilot level, are presented by Palo et al. [22]. The results achieved showed that membrane reactors can be effectively used in all the mentioned applications. It should be noted, however, that in most of the proposed solutions, the concept of the membrane reactor is based on the application of a sequence of reaction–separation–reaction units rather than on the application of a reactor in which the catalyst and membrane are present in the same process unit, according to the concept of process intensification. This is driven by particular cases where there is a mismatch between optimal operating conditions for catalyst and membrane operation, thereby also impacting on the operation and maintenance of the reaction system. One specific case of this is, for example, the dehydrogenation of propane, where coke formation on the surface of Pd-based membranes prevents continuous operation of an integrated membrane DH process at the conventionally applied temperatures required for the catalysts, i.e., 500–600 °C [23]. 

Pd-based membranes have also been applied in hydrogen and chemical heat storage systems, in small and medium scale, from renewable energy. Conventional storage solutions store hydrogen physically, by compression or liquefaction, to increase the low volumetric energy density compared to atmospheric conditions. Liquid organic hydrogen carriers (LOHCs), on the other hand, propagate the reversible chemical binding of hydrogen to an organic liquid. The LOHC can store 6.2 wt% hydrogen when fully loaded, which corresponds to a storage density of 17.5 L_LOHC_/kg_H2_. In general though, dehydrogenation is technically more difficult to implement than hydrogenation. As a solution, Pd-based membrane reactors have already been applied to facilitate the dehydrogenation of LOHC methylcyclohexane with promising results. For example, Wunsch presents an intensified LOHC-dehydrogenation, applying a multi-stage microreactor and Pd-Based membrane process design, in this Special Issue of *Membranes* [6,16]. Simulations were carried out, which showed that the described approach can drastically intensify the whole dehydrogenation process, in addition to the in situ purification of the hydrogen.

## 3. Final Remarks

Overall, the editors are convinced that metal membranes, and Pd-based membranes in particular, have a lot more to contribute than what has already been demonstrated world-wide. We hope that readers enjoy this Special Issue and gain inspiration from it for their own work. In the end, technological changes are the fruit of ideas planted as seeds in researchers’ minds: the more that individual minds are connected to each other, the higher the probability of creating originality. In this sense, this Special Issue represents a small attempt to increase the connectivity among interested minds, as we provide our contributions to technological innovation.
